# Amorphous In–Ga–Zn–O Powder with High Gas Selectivity towards Wide Range Concentration of C_2_H_5_OH

**DOI:** 10.3390/s17061203

**Published:** 2017-05-24

**Authors:** Hongxiang Chen, Wei Jiang, Lianfeng Zhu, Youwei Yao

**Affiliations:** Advanced Materials Institute, Graduate School at Shenzhen, Tsinghua University, Shenzhen 518055, China; chx14@mails.tsinghua.edu.cn (H.C.); jw15@mails.tsinghua.edu.cn (W.J.); zhulianfeng@gmail.com (L.Z.)

**Keywords:** a-IGZO, gas sensor, ethanol, H_2_, CO, selectivity

## Abstract

Amorphous indium gallium zinc oxide (a-IGZO) powder was prepared by typical solution-based process and post-annealing process. The sample was used as sensor for detecting C_2_H_5_OH, H_2_, and CO. Gas-sensing performance was found to be highly sensitive to C_2_H_5_OH gas in a wide range of concentration (0.5–1250 ppm) with the response of 2.0 towards 0.5 ppm and 89.2 towards 1250 ppm. Obvious difference of response towards C_2_H_5_OH, H_2_, and CO was found that the response e.g., was 33.20, 6.64, and 2.84 respectively at the concentration of 200 ppm. The response time and recovery time of was 32 s and 14 s respectively towards 200 ppm concentration of C_2_H_5_OH gas under heating voltage of 6.5 V.

## 1. Introduction

Ethanol (C_2_H_5_OH) is a kind of volatile, flammable, and explosive gas. Monitoring C_2_H_5_OH gas is of great importance for industrial safety or preventing traffic accidents from drunk driving [1]. Therefore, the research of C_2_H_5_OH gas sensors of high sensitivity, high selectivity, and fast response/recovery speed is practical significant. Because of their high sensitivity and low-cost fabrication, semiconductor metal oxide (SMO) gas sensors such as SnO_2_, ZnO, etc. have attracted considerable interest, and are widely used for detecting gases [2,3,4,5,6,7,8].

Most of the SMO sensors are consisted of polycrystalline oxides. Different from the case of the polycrystalline SMO, there is no grain boundary network in amorphous oxide semiconductor (AOS) [9]. However, there is a conduction band and forbidden band in the energy band structure of AOS, which is similar to that of polycrystalline SMO. Therefore, AOS with proper conductivity also serves as a promising gas-sensing system for further in-depth studies.

Among the AOSs, an n-type semiconductor, amorphous indium gallium zinc oxide (a-IGZO) is widely used in the field of LCD displays as transparent thin-film transistors (TFTs) because of its high carrier mobility (>10 cm^2^/Vs) [10]. It is thought that the high carrier mobility originates from the formation of a conduction band minimum in the spherical s-orbitals of the post-transition-metal cations [11,12]. The low ability for those cations to attract electrons in their spherical s-orbitals causes the electron transportation to occur easily, which makes a-IGZO material sensitive to environmental conditions, especially the redox property of atmosphere.

It has been reported that the response of amorphous-InGaZnO_4_ (a-IGZO_4_) thin film towards reducing gas H_2_ in a range of concentration between 0.5 ppm and 50 ppm, (e.g., response is 0.7 at 350 °C for 12.5 ppm, response time is around 200 s; recovery time is around 350 s) and oxidizing gases NO_2_ (e.g., response is 33 at 200 °C for 5 ppm, response time is around 650 s; recovery time is around 1250 s) [9]. Another example is that a-IGZO thin film was used to detect ozone gas with concentrations ranging from 35 to 168 ppb at a resolution of about 20 ppb with light-emitting diodes to enhance. The response time and recovery time of 90% (the time for response increasing by the 90% of the difference response value and the time for response decreasing by the 90% of the difference response value) was 235 s and 327 s, respectively [13,14]. Meanwhile, a kind of a-IGZO gas sensor with long-term stability showing around 6% variation of gas sensitivity was reported to detect 750 ppm of acetone. The T_37-res_ and T_37-rec_ was 37 s and 53 s respectively [15]. All of the a-IGZO gas-sensing devices above were made to be a thin film to detect target gas and some of them respond to some particular kinds of gas in a considerably low concentration such as ozone. However, the response time and recovery time of those devices still remain several minutes.

A solution-based process is also one of the most promising strategies for obtaining a-IGZO because of its low price, and moderate and controllable reaction condition [12,16]. To date, there has been no report on the gas-sensing performance of a-IGZO powder prepared by solution-based process and post-annealing process. Thus, we focus here on detecting reducing gases (C_2_H_5_OH, H_2_, CO) with a-IGZO powder prepared by a typical solution-base process. 

## 2. Experimental Section

### 2.1. Preparation of IGZO

Indium nitrate hydrate (In(NO_3_)_3_·H_2_O, 99.9%), gallium nitrate hydrate (Ga(NO_3_)_3_·3H_2_O, 99.9%), zinc acetate dehydrate (ZnOAc_2_·2H_2_O, 99%) 2-methoxyethanol (2-ME), monoethanolamine (MEA), were bought from Aladdin (Shanghai) Industrial Inc., and used as received without further purification. The typical process of fabrication is as follows [17]. 0.549 g In (NO_3_)_3_·H_2_O, 0.639 g Ga(NO_3_)_3_·3H_2_O, and 0.751 g ZnOAc_2_·2H_2_O as sol-gel precursors were used dissolved in 25.0 mL (2-ME) and 1.0 mL (MEA) as a stabilizer. Stirred for 1 h at 60 °C and dried for one day at 50 °C to form the IGZO sol. The IGZO sol was annealed at 450, 600, and 700 °C for 3 h to produce IGZO powders. 

### 2.2. Gas-Sensing Characterization

Typical fabrication and structure of the sensor device can be found in our early work [2]. The IGZO powder was grounded with deionized water to form slurry. The slurry was coated onto a piece of alumina substrate whose front side had a pair of gold electrodes printed on and whose backside had a Ru_2_O_3_ heater printed on. The sensor was placed into a sealed container with 16 L volume. An air fan in the container helps the gas quickly spread. Certain amounts of target gas were injected into container mixing with the air with 40% humidity at 25 °C as carrier gas to modulate gas concentration. The ethanol gas was the gasified ethanol droplet injected into the container by a microsyringe since the evaporation speed of ethanol is rather fast. The concentration of the ethanol gas depends on the accurate amount of the ethanol droplet according to Avogadro’s law. Different heating voltages were applied to the Ru_2_O_3_ heater so that the gas sensor can work at different operation temperatures. The alumina substrate was about 250 °C at the heating voltage of 5.0 V. The resistance between the gold electrodes changes when target gas was injected into the container. A multimeter (Victor 86B) was used to collect electrical current (I). The resistance (R) was calculated by a certain bias voltage of 5.0 V.

The sensor response is defined as followed: S = (I_g_ − I_a_)/I_a_ or S = (R_a_ − R_g_)/R_g_, where I_a_ and R_a_ is stable current and resistance obtained when the sensor is exposed in the air; I_g_ and R_g_ is stable current and resistance obtained when the sensor is exposed in the target gas. In order to represent the speed of response and recovery of the sensor, we define T_90-res_ as the time for resistance to increase by 90% (R_a_ – R_g_) and the T_90-rec_ is the time for resistance to decrease by 90% (R_a_ – R_g_).

### 2.3. Characterization

X-ray diffraction (XRD) patterns were taken with an X-ray diffractometer (D/max 2500/PC, RIGAKU Corp., Tokyo, Japan) using Cu K_α_ radiation (*λ* = 1.5406 Å). Radiation operated at 40 kV and 200 mA was applied. The scan speed is 4°/min. The morphologies of the final product were investigated by a scanning electron microscope (SEM, Hitachi Limited S-4800, Tokyo, Japan) and a field emission transmission electron microscope (FETEM, FEIG2F30, 300 Kv, Hillsboro, OR, USA). FT-IR spectroscopy was performed using a Nicolet 6700 Fourier transform spectrometer (Waltham, MA, USA) at a resolution of 4 cm^−1^ and 32 scans/spectrum. The powder sample was pressed into a self-supporting disc (ca. 20 mm in diameter), and placed into an in situ stainless steel IR cell with removable KBr windows and a thermocouple used for monitoring the temperature. Different temperature from RT to 500 °C and different atmosphere can be applied to the cell. Prior to dosing target gas, the sample was heated at 500 °C under N_2_ for 30 min to get clean surface. Firstly, the spectra of the sample was measured the transmittance under the synthetic air (80% O_2_ and 20% N_2_, 100 mL/min of total gas flow) at temperatures of 30, 100, 200, 300, and 400 °C as the background spectra. Then IR adsorption involved 1000 ppm target gas was measured on the corresponding experiment condition.

## 3. Result and Discussion

### 3.1. Morphology of the Final Product

**Figure 1:**
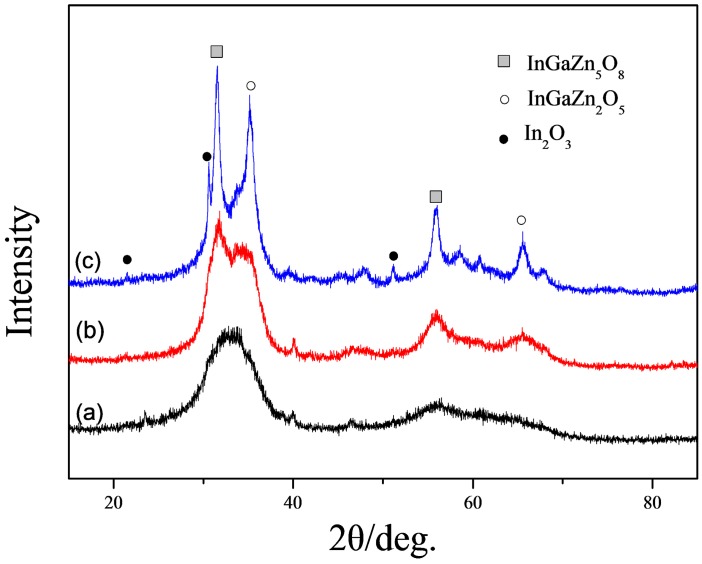
XRD patterns of IGZO powder annealed at (**a**) 450 °C; (**b**) 600 °C; (**c**) 700 °C.

Figure 1a–c shows the XRD patterns of IGZO powder after annealing at (a) 450 °C; (b) 600 °C; (c) 700 °C. Few obviously sharp diffraction peaks and a wide diffraction band from 35° to 40° were observed in Figure 1a which indicates that the powder retains an amorphous status. As the annealing temperature increasing, the shaper peaks of diffraction observed because of crystallization. The XRD pattern of the powder annealed at 700 °C, shown in Figure 1c, indicates the presence of crystals. The d values of 0.41, 0.29, and 0.18 nm, corresponding to the diffraction peaks of 21.49°, 21.59°, 51.02° are approximately equal to the d values of 0.413, 0.292, and 0.178 nm, corresponding to the (211), (222), (440) of In_2_O_3_ (JCPDS No. 06-0416); The d values of 0.28 nm and 0.16 nm, corresponding to the diffraction peaks of 31.51° and 55.96° are equal to the d values of 0.283 nm and 0.164 nm, corresponding to the (101), (110) of InGaZn_5_O_8_ (JCPDS No. 40-0255). The d values of 0.25 nm and 0.14 nm, corresponding to the diffraction peaks of 30.59° and 65.58° are equal to the d values of 0.254 nm and 0.142 nm, corresponding to the (104) and (201) of InGaZn_2_O_5_ (JCPDS No. 40-0252).

**Figure 2:**
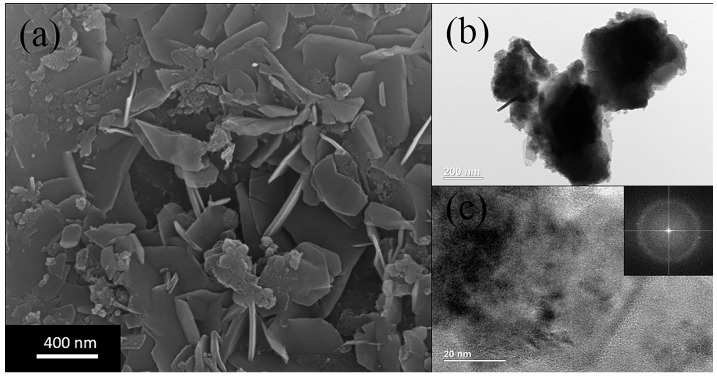
(**a**) SEM image, (**b**,**c**) TEM image of IGZO powder annealing at 450 °C.

The SEM image in Figure 2a shows a clear picture of plate morphology information. The size of one layer is about 400 nm. The TEM image in Figure 2b,c shows that there are few obvious lattice fringes observed. The discrete diffraction rings observed in FFT image also indicate that the IGZO powder remains amorphous phase in the thin plates which is corresponding to the XRD diffraction information above.

**Figure 3:**
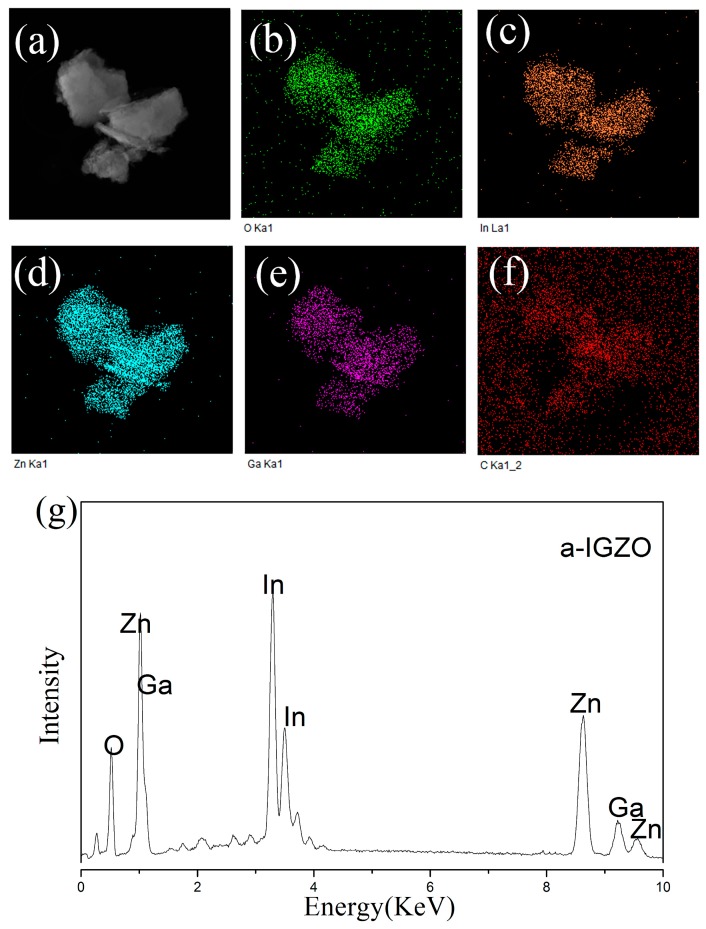
EDS image of IGZO powder annealing at 450 °C, (**a**) dark field image of TEM, distribution image of (**b**) O; (**c**) In; (**d**) Zn; (**e**) Ga; (**f**) C; (**g**) atomic analysis of EDS.

The EDS image shown in Figure 3 also shows the existence of the homogenous distribution of the metal elements of indium, gallium, and zinc. The MEA stabilizer may play an important role to solve the problem of concentration uniformity. The atomic ratio of the a-IGZO powder, analyzed by EDS, was found to be In:Ga:Zn = 1.3:1.0:3.3 as shown in Figure 3g.

### 3.2. Gas-Sensing Characterization

**Figure 4:**
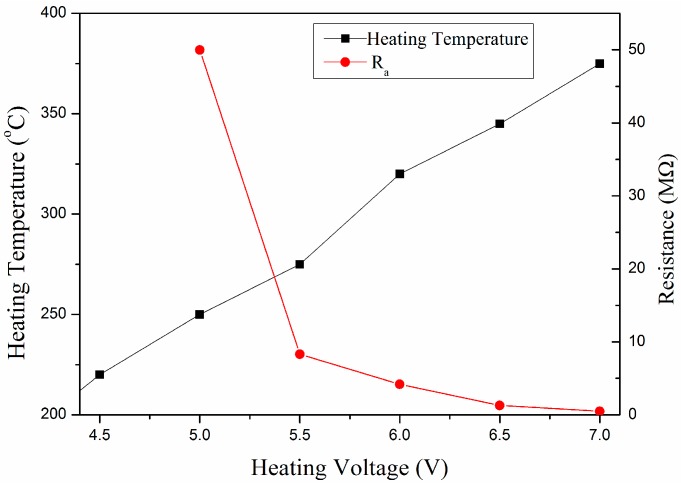
R_a_ of a-IGZO powder under different heating temperature.

The gas-sensing performance of the IGZO powder annealed at 450 °C was investigated. Table 1, Table 2 and Table 3 show the gas-sensing properties towards 200 ppm C_2_H_5_OH, H_2_ and CO. Figure 4 shows the R_a_ of a-IGZO powder at different operating temperature. When the heating voltage (HV) was 5.0 V (ca. 250 °C), Ia was 0.1 μA, the corresponding stable resistance Ra was 50 MΩ. The resistance Ra decreased with the increasing heating voltage. When the heating voltage was 7.0 V (ca. 375 °C), the resistance Ra decreased 100 times to 0.5 MΩ. When the sensor is exposed to the reducing gas, the current increases to Ig, accordingly, the resistance decreases to the Rg. To 200 ppm CO, the response mainly remained at 1.5; to 200 ppm H_2_, the response was 7.1 at the operating temperature of 275 °C and 320 °C, 2.5 at 375 °C; to 200 ppm C_2_H_5_OH, the highest response was 43 at 250 °C.

As for the T_90-res_ and T_90-rec_, T_90-res_ was 2~4 s and the T_90-rec_ was about 12 s for 200 ppm CO; T_90-res_ was 4~5 s and the T_90-rec_ was about 12 s for 200 ppm H_2_; T_90-res_ was about 50 s; and the T_90-rec_ was about 25 s for 200 ppm C_2_H_5_OH, the reason that the T_90-res_ towards C_2_H_5_OH is longer those that towards H_2_ and CO may be the evaporation process of C_2_H_5_OH droplet. On previous reports, the response time and recovery time of gas-sensing devices fabricated based on a-IGZO thin film are 237–1250 s [9,13,15]. Thus, this work makes an improvement on the speed of response and recovery. It is thought that the absorption and desorption kinetics is associated with the nature of the oxide, the concentration of the gas, and the operating temperature [18]. In addition, the status of the oxide surface may be a critical factor influencing the speed of response and recovery. Compared to the a-IGZO made to be the thin film, in our work, the a-IZGO powder arranges in a looser way. The surface status of a-IGZO powder was more comprehensive, including more interspace and dangling bands, which may lead a fast response and recovery speed.

**Figure 5:**
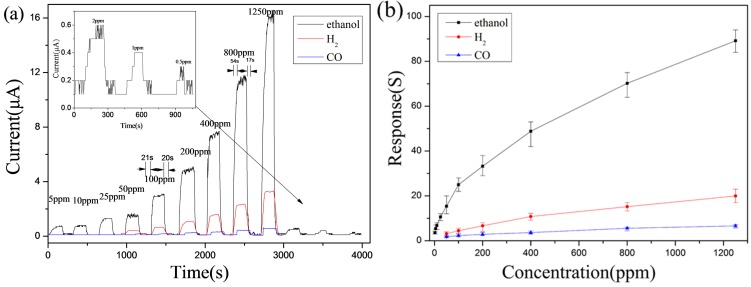
(**a**) Responses of the sensor based on a-IGZO powder annealed at 450 °C exposed towards C_2_H_5_OH, H_2_, and CO from 0.5 ppm to 1250 ppm (**b**) their corresponding plots of sensitivities versus concentrations.

The HV with the highest sensitivity for each kind of gas (e.g., 5.0 V for CO; 6.5 V for C_2_H_5_OH; 5.5 V for H_2_) was chosen to investigate the gas-sensing performance with different levels of gas concentration (0.5–1250 ppm for C_2_H_5_OH; 50–1250 ppm for H_2_ and CO). From Figure 5a,b, it can be seen that a-IGZO shows repeatable responses to C_2_H_5_OH, H_2_, and CO. Besides, obvious difference of response towards C_2_H_5_OH, H_2_, and CO was also found. For example, the response towards 1250 ppm C_2_H_5_OH, H_2_, and CO was 89.2, 20.2, 6.6 respectively; the response towards 200 ppm was 33.2, 6.6, and 2.8, with the ratio equally to 11.7: 2.3:1.0, which shows great selectivity of the as-prepared a-IGZO powder. What is more, gas-sensing performance towards low concentration of C_2_H_5_OH (0.5–50 ppm) is also measured. This a-IGZO was found to be highly sensitive to C_2_H_5_OH gas in a wide range of concentrations (0.5–1250 ppm) with the response of 89.2 towards 1250 ppm and 2.0 towards 0.5 ppm.

### 3.3. In Situ FT-IR

**Figure 6:**
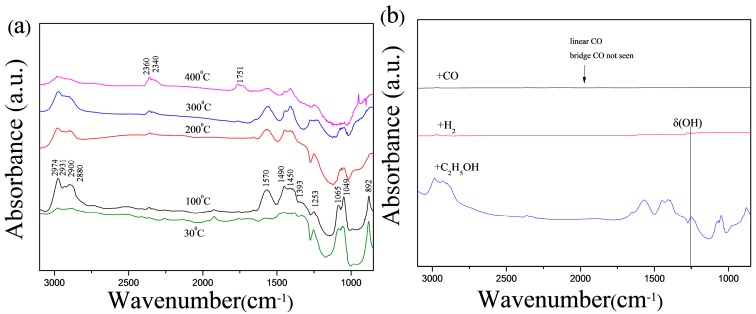
FT-IR spectra of (**a**) a-IGZO powder absorbed with C_2_H_5_OH vapor at 30 °C and subsequently heating to 100, 200, 300, and 400 °C; (**b**) a-IGZO powder absorbed with C_2_H_5_OH, CO, H_2_ vapor at 100 °C.

It is helpful to explore the gas-sensing properties by investigating the absorption ability of gas [2]. The in situ FT-IR spectra of C_2_H_5_OH absorbed a-IGZO powder was collected at the temperature of 30, 100, 200, 300, and 400 °C. The evidence of C_2_H_5_OH physisorption and chemisorption are observed. The peaks at 1253, 1049, and 892 cm^−1^ can be assigned to δ (OH), υ (OC), and υ (CCO), which can be attributed to the vibrational modes of C_2_H_5_OH absorbed on the surface. C_2_H_5_OH species on various metal oxides have been observed [19]. The characteristic peaks at 2974, 2931, 2900, 2880, 1450, 1393, 1350, and 1065 cm^−1^ found in Figure 6a correspond to absorbed ethoxide species. The assignments of the ethoxide peaks are listed in Table 4 with various catalysts reorts [20,21,22,23]. The absorbance of those absorption peaks listed above is observed to be the highest at 100 °C and declines with increasing temperature, which shows that the amount of the absorbed C_2_H_5_OH on a-IGZO powder is the largest at 100 °C, and decrease with the increasing temperature. Two peaks at 1570 cm^−1^ and 1490 cm^−1^ can be assigned to υ_a_ (OCO), υ_s_ (OCO) which can be attributed to the vibrational modes of acetate structure [20,24]. Band with peak at ca. 1751 cm^−1^ attributed to (C=O) of acetaldehyde species appeared at 300 °C, and whose intensity increased with temperature increasing, which shows the process of dehydrogenation and oxidation taking place [25,26]. While there is no obvious typical absorption band at 2120 cm^−1^ (linear CO), 1916 cm^−1^ (bridging CO) found when a-IGZO powder was exploded in CO atmosphere [19]. It may indicate that the process of CO and H_2_ absorption is not as easy as C_2_H_5_OH absorption. The feature corresponds to high sensitivity and selectivity of C_2_H_5_OH. However, in addition to the gas adsorption behaviors, the mechanism of selectivity is very complex and depends on many other factors such as catalytic activity and acid-base properties of the sensor [27].

As an n-type semiconductor, a-IGZO with proper conductivity serves as a gas-sensing system, responds to reducing gas (C_2_H_5_OH, H_2_, and CO). The chemisorbed oxygen species generates an electron depletion layer on the surface of the a-IGZO powder. When the device is exposed to the air, the oxygen molecules from the air are chemisorbed on the surface, the oxygen attracts electrons from the conduction band of a-IGZO powder. As a result, the density of the carrier decreased, the resistance of the sensor increased [28,29,30]. When the device is exposed to the reducing target gas, the gas reacts with the chemisorbed oxygen and the electron from reduce gas molecular transported to the conduction band. Thereby, the resistance of the sensor decreases. In this work, the IGZO powder annealing at 450 °C retains an amorphous status, different from the case of crystalline, most atoms of a-IGZO are not arranged in well-organized positions, and the arrangement of its internal atoms is relatively disordered. Nonetheless, the spherical s-orbital of the post-transition cation is large enough so that even if there is a distortion of the metal-oxygen-metal band, the carriers are able to transport through the overlap direct between the neighboring metallic s-orbitals [11].

## 4. Conclusions

In summary, amorphous indium gallium zinc oxide (a-IGZO) powder was prepared by a typical solution process and post-annealing process at 450 °C, which was found to be highly sensitive to C_2_H_5_OH gas in a wide range of concentration (0.5–1250 ppm) with the response being that 89.2 towards 1250 ppm and 2.0 towards 0.5 ppm. The selectivity of IGZO powder with the response to C_2_H_5_OH, H_2_, and CO at a concentration of 200 ppm was 33.2, 6.64, and 2.84, the ratio was 11.7:2.33:1. Fast response time (T90-res, 2–67 s) and recovery time (T90-rec, 10–28 s) were also measured.

## Figures and Tables

**Table 1:** Gas-sensing performance of IGZO gas sensor (450 °C) with different heating voltage (CO-200 ppm).

HV (V)	HT (°C)	Ia (μA)	Ig (μA)	S	T_90-res_ (s)	T_90-rec_ (s)
5.0	250	0.3	0.8	1.6	2	10
5.5	275	0.8	2.0	1.5	4	12
6.0	320	1.6	3.3	1.1	4	10
6.5	345	3.3	7.8	1.4	4	12
7.0	375	10.0	26.0	1.6	2	12

**Table 2:** Gas-sensing performance of IGZO gas sensor (450 °C) with different heating voltage (C_2_H_5_OH-200 ppm).

HV (V)	HT (°C)	Ia (μA)	Ig (μA)	S	T_90-res_ (s)	T_90-rec_ (s)
5.0	250	0.1	4.4	43	54	28
5.5	275	0.6	6.2	9.3	51	21
6.0	320	1.2	15.2	11.7	67	27
6.5	345	3.8	26.7	6.0	32	14
7.0	375	\	\	\	\	\

**Table 3:** Gas-sensing performance of IGZO gas sensor (450 °C) with different heating voltage (H_2_-200 ppm).

HV (V)	HT (°C)	Ia (μA)	Ig (μA)	S	T_90-res_ (s)	T_90-rec_ (s)
5.0	250	0.3	1.8	5.0	5	13
5.5	275	0.7	5.7	7.1	5	12
6.0	320	1.6	13.0	7.1	4	12
6.5	345	8.0	33.0	3.1	4	12
7.0	375	10.0	35.0	2.5	4	12

**Table 4:** IR Vibrational frequencies and mode assignment for ethoxide species on IGZO and various metal oxides.

Mode	Al_2_O_3_ [20]	TiO_2_ [21]	Pt/CeO_2_ [22]	Rh/CeO_2_ [23]	Sb-SnO_2_ [2]	IGZO (This Work)
υ_as_ (CH_3_)	2970	2971	2977	2981	2977	2974
υ_as_ (CH_2_)	2930	2931	2933	2934	-	2931
υ_s_ (CH_3_)	2900	-	2912	2911	2907	2900
υ_s_ (CH_2_)	2870	2870	2878	2878	-	2880
δ_as_ (CH_3_)	-	1473	1480	1478	-	-
δ_as_ (CH_3_)	1450	1447	1451	1450	-	1450
δ_s_ (CH_3_)	1390	1379	1399	1399	1405	1393
δ_s_ (CH_3_)	-	-	-	-	-	-
ω_as_ (CH_3_)	-	1356	-	-	-	1350
υ (OC)_mono-_	1115	1119	1081	1080	-	-
υ (OC)_bi-_	1070	1042	1037	1038	1068	1065
